# Analysis of Modern Optical Inspection Systems for Parts Manufactured by Selective Laser Melting

**DOI:** 10.3390/s20113202

**Published:** 2020-06-04

**Authors:** Sara Giganto, Susana Martínez-Pellitero, Eduardo Cuesta, Víctor M. Meana, Joaquín Barreiro

**Affiliations:** 1 Department of Mechanical, Computer and Aerospace Engineering, Universidad de León, Campus de Vegazana, 24071 León, Spain; sgigf@unileon.es (S.G.); jbarg@unileon.es (J.B.); 2 Department of Construction and Manufacturing Engineering, University of Oviedo, Campus de Gijón, 33204 Gijón, Spain; ecuesta@uniovi.es (E.C.); meanavictor@uniovi.es (V.M.M.)

**Keywords:** optical measurement systems (OMSs), dimensional and geometrical accuracy, metrological comparison, non-contact inspection, 3D scanning, additive manufacturing (AM), selective laser melting (SLM)

## Abstract

Metal additive manufacturing (AM) allows obtaining functional parts with the possibility of optimizing them topologically without affecting system performance. This is of great interest for sectors such as aerospace, automotive, and medical–surgical. However, from a metrological point of view, the high requirements applied in these sectors constitute a challenge for inspecting these types of parts. Non-contact inspection has gained great relevance due to the rapid verification of AM parts. Optical measurement systems (OMSs) are being increasingly adopted for geometric dimensioning and tolerancing (GD&T) verification within the context of Industry 4.0. In this paper, the suitability (advantages and limitations) of five different OMSs (based on laser triangulation, conoscopic holography, and structured light techniques) for GD&T verification of parts manufactured by selective laser melting (SLM) is analyzed. For this purpose, a specific testing part was designed and SLM-manufactured in 17-4PH stainless steel. Once the part was measured by contact (obtaining the reference GD&T values), it was optically measured. The scanning results allow comparing the OMSs in terms of their inspection speed as well as dimensional and geometrical accuracy. As a result, two portable systems (handheld laser triangulation and structured blue-light scanners) were identified as the most accurate optical techniques for scanning SLM parts.

## 1. Introduction

Additive manufacturing (AM) is an emerging process to make objects from a 3D CAD model, joining materials layer by layer. AM processes include different techniques, materials, and equipment, and they have greatly evolved in recent years due to the significant advantages compared to subtractive manufacturing processes: complex geometry manufacturing with high precision, material savings, design flexibility, parts customization, and integrating new applications thanks to the continuous development of new materials and additive systems [1]. Advances in AM are generating new design possibilities, products, and production paradigms. The introduction of new business models and the general time-to-market reduction are driving interest in AM technologies [2].

Metal AM allows the manufacturing of final topological optimized parts, improving the performance of the system. It enables designers to create lightweight parts and minimize material usage. These factors offer great benefits in sectors with high requirements, such as automotive, aerospace, or medical–surgical. In these types of industries, studies are being carried out to make AM technologies more versatile and safer using its ability to revolutionize global manufacturing and distribution of parts, allowing very high customization with better cost and energy consumption [3].

Among AM technologies [4], powder bed fusion (PBF) is the category encompassing the selective laser melting (SLM) technique, among others. In this process, powder particles are selectively fused, layer by layer, by a thermal source. SLM allows the fusion and solidification of metallic powder using a laser beam to manufacture small and medium-sized metal parts [5,6]. Parts manufactured by this technique may have internal defects [7], surface texture [8,9], and/or geometrical and dimensional errors. The SLM process produces complex thermal cycles that result in residual stresses on the manufactured part, which deforms it [10]. The stress and deformation distribution are also influenced by the scanning strategy, which is another variable in the printing process [11]. These factors, among others, negatively affect the dimensional accuracy of the parts [12], which limits the application to functional parts in the aerospace and automotive sectors.

Due to the high requirements of these sectors, metrological inspection acquires great importance. However, from the point of view of metrology, the characteristics of the AM parts are challenging. The freedom of design that AM offers compared to subtractive technologies requires more complex measurement techniques and greater data processing capabilities. In addition, it is necessary to integrate metrology (whose main purpose is to detect early problems during manufacturing) into current SLM machines and processes to facilitate marketing the resulting products in less time [13]. Some researchers have focused on the study of dimensional errors caused by deformations resulting from the printing process. Online inspection, specifically in a machine, can help to know and reduce this type of error. Kalms et al. [14] proposed an online evaluation of the laser beam melting (LBM) process layer by layer for both the printed part and the powder deposition using structured light (SL) systems. He et al. [15] focused their study on the in situ deformation characterization produced by PBF techniques using optical systems. Everton et al. [16] reviewed the visual and camera-based systems developed so far to monitor AM processes in order to understand them, rather than to identify material discontinuities. Process monitoring methods allow ensuring manufacturing integrity. Cordero et al. [17] compared two non-contact measurement techniques (pyrometer and infrared thermography) to monitor layerwise surfaces of a PBF process and identify manufacturing defects, such as porosity, warping, and delamination. These types of process monitoring methods ensure the manufacturing integrity of a part but not its dimensional quality.

Likewise, the surface texture of SLM metal parts, characterized by a typical high roughness, challenges existing measurement and characterization techniques. Some researchers have focused their efforts on surface metrology. Townsend et al. [18] made a very complete review of non-contact systems used for the surface metrological analysis of AM parts by comparing them with contact systems. In another investigation, Townsend et al. [19] designed a series of surface-specific measurement test artifacts for verifying AM processes. This study proposes a configuration of part-building orientation based on the surface texture information obtained. Newton et al. [20] studied the suitability of focus variation (FV) equipment for analyzing the surface topology of parts made by electron beam melting (EBM) and LBM.

In this context, commercial inspection systems have improved their precision in recent years. Contact systems can measure prismatic parts and freeform surfaces on SLM parts with high accuracy, but they are relatively slow and only allow acquiring a limited number of points on a surface. Non-contact inspection is especially relevant for quick verification of AM parts. Optical measurement systems (OMSs) provide dense point clouds in very short times, reducing inspection cycles. Different non-contact techniques use lasers, structured white/blue light, or photogrammetry to capture a point cloud without touching the part surface. Stavroulakis and Leach [21] reviewed optical systems to measure AM parts. These systems, integrated into coordinate measuring machines (CMM), machine-tools, or portable coordinate measuring arms (AACMM or CMA), are increasingly adopted for dimensional and geometrical verification in different sectors within the context of Industry 4.0 [22]. Selecting the most appropriate optical inspection system depends on part design specifications, geometry, size, and/or accessibility, among others. With regard to the accuracy of the inspection operation, it depends on several factors. Some factors are directly related to the scanning strategy, such as the orientation and/or position of the sensor with respect to the digitized surface, the number of scans necessary to achieve the complete digitization of a part or surface, and the configuration parameters of the sensor itself. Other factors are related to the characteristics of the working part, such as its geometry, texture, or color. Several studies have demonstrated the influence of these factors in the process of digitization and accuracy according to different non-contact systems. Gerbino et al. [23] analyzed the influence of some scanning factors in the measurement process with a laser scanning (LS) sensor. In a previous work, we studied the accuracy of a structured blue-light (SL) sensor when measuring different form features under usual working conditions with different fields of view (FOVs) [24]. Dury et al. [25] verified the inspection accuracy by measuring surface finishes common to precision engineering and promoted best practices when performing 3D optical scanner measurements. Yue et al. [26] analyzed the influence of surface reflectivity on the precision of SL metrology. Rico et al. [27] studied the influence of surface roughness on scanning quality with a commercial type of conoscopic holography (CH) sensor integrated into a CMM.

In this work, we study the suitability of different active OMSs for dimensional and geometrical verification of parts manufactured by the SLM process. In particular, we compare five different active OMSs: a laser triangulation sensor on a CMM (LS-CMM), a laser triangulation sensor integrated into a portable CMA (LS-CMA), a portable and handheld laser triangulation scanner (HLS), an SL, and a CH. In addition to analyzing the different optical systems, the study also includes a comparison between stationary and portable systems. As is well known, portable systems have less accuracy but offer advantages in terms of speed and field of application, since they can be used anywhere without the requirement to be in a laboratory with special conditions. Cuesta et al. [28] analyzed the suitability to inspect parts using a laser sensor incorporated in a CMA, measuring different geometrical features and comparing them with the results obtained using a contact system on a CMM, used as a reference. Mian and Al-Ahmari [29] studied the suitability of contact and non-contact digitizing sensors mounted on a stationary and a portable CMA.

LS sensors are currently used both in reverse engineering (RE) applications and in inspecting parts. Among them, laser triangulation sensors are the most widely used in metrological applications. This type of sensor can be integrated into a CMM or a CMA, as well as being perfectly portable and handheld.

SL scanners use different light patterns projected onto the part to capture many point clouds in very short times. This technology is adequate for diverse applications in industry, as well as RE and inspection tasks.

CH is a technique that uses an incoherent-light interferometrically thanks to the refraction properties of uniaxial crystals. Álvarez et al. [30] showed several industrial applications with this type of sensor and made a comparison with an LS sensor based on triangulation. The CH system offers simplicity, high stability, and precision for optical metrology. Although these researchers initially focused their work on the online analysis of roughness, they demonstrated the CH suitability to be applied in macrosystems by adjusting the appropriate parameters.

The objective of this paper is to analyze the advantages and disadvantages of portable optical systems, comparing them with traditional stationary OMSs, in the scope of SLM parts inspection. Our goal is to define the advantages and limitations of these optical systems, and their suitability for measuring metal SLM parts. We designed a test part based on limitations and characteristics of both the SLM process and SLM machines, adequate for measuring with contact and non-contact metrological systems. Other researchers have reported comparative studies with different OMSs oriented towards RE. Mian and Al-Ahmari [29] proposed the implementation of multi-criteria decision-making methods for identifying the most appropriate digitization equipment. Ramos and Santos [31], using simple test parts, compared different scanning systems by reconstructing the surfaces from the point clouds obtained in the digitizing process. They compared the quality and dispersion of both the point distributions and their distribution in three calibrated models. Ameen et al. [32] identified the capabilities and limitations of two different handheld scanners by comparing accuracy, scanning time, triangle numbers, ease of use, and portability. This study was oriented towards large parts in the automotive industry. Tóth and Živčák [33] evaluated two different scanners, both dimensionally and geometrically, by comparing the obtained point clouds against the part’s nominal CAD. In this study, the test part was manufactured additively in a white material that optically facilitates point digitization. Iuliuano and Minetola [34] compared an LS sensor with an SL sensor to digitize the sculptural parts. The comparison was carried out based on criteria such as accuracy, scanning speed, robustness, ease of use, transportability, and cost/performance ratio. The results were based on the level of detail achieved when digitizing, with both systems, a test part manufactured additively, and turned out to be a qualitative comparison. Guerra et al. [35] analyzed the performance of three optical scanners to measure a miniature step gauge manufactured additively from polyphenylene sulfide (PPS). The SL system was the most suitable for this type of part and process. Rivas et al. [36] defined “design-for-metrology as a methodology in which the user aims to optimize the part measurement and quality control processes and facilitate fast and accurate measurements on AM parts”. In particular, they developed an artifact for high-speed sintering. They used several techniques, such as a traditional CMM, close-range photogrammetry, and X-ray computed tomography.

Unlike the aforementioned research, our work allows comparing several optical systems for application in SLM processes with high-quality requirements. Therefore, this study includes both the dimensional and geometrical evaluation of a specifically designed test part using the point clouds obtained with five OMSs. This comparative study was carried out using the Geomagic^®^ Control™ software.

## 2. Materials and Methods

**Figure 1:**
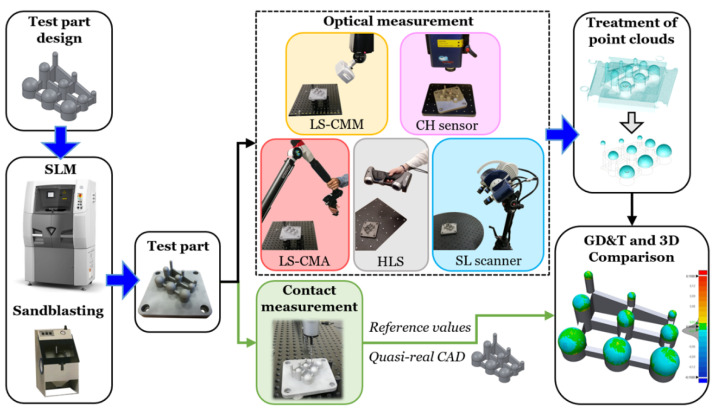
Methodology evaluating a selective laser melting (SLM) test part using different optical systems.

Below is a summary of the methodology followed for evaluating an SLM metal part using different OMSs. Figure 1 shows the material and methods used. The five main steps of the methodology are detailed in the following sections.

1. Test part design: A test part was designed considering the restrictions of the AM machine and process (allowing the characterization of its limits) and the requirement for measuring with contact and optical systems.
2. SLM manufacturing process and sandblasting post-process: Once the CAD design was exported in *.stl format, the test part was manufactured in 17-4PH stainless steel using SLM. The part was post-processed by sandblasting to improve the surface finish and facilitate the acquisition of the point clouds with several OMSs.
3. Contact measurement: The test part was measured with high precision using a CMM, obtaining the geometric dimensioning and tolerancing (GD&T) model. These contact measurements were taken as reference or quasi-real CAD values.
4. Optical measurement: The sphere-based test part was digitized using different OMSs (laser triangulation, CH, and SL sensors).
5. Treatment of point clouds and GD&T measurements: The point clouds were cleaned by removing all scanned points not belonging to the part spheres. Subsequently, the GD&T measurements and 3D comparisons were performed between the quasi-real CAD and the point clouds obtained by the different non-contact systems.

### 2.1. Test Part Design

**Figure 2:**
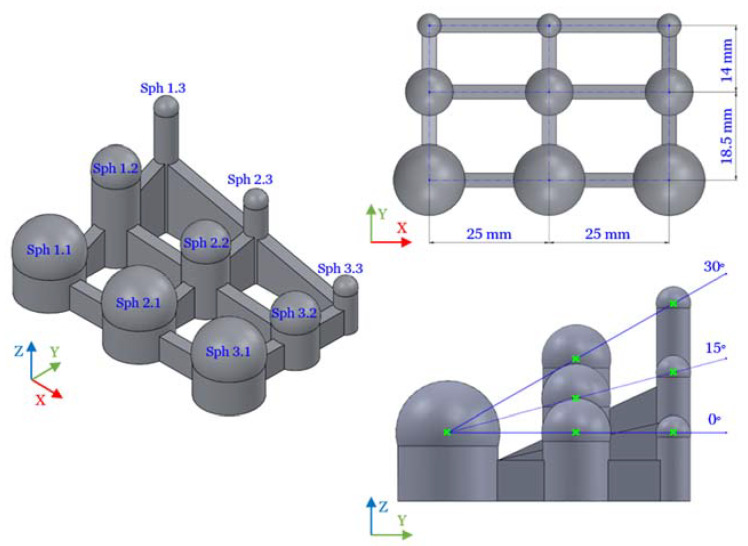
Test part CAD: sphere designations and design features.

Both contact and non-contact measuring systems and the manufacturing technology (SLM) were considered for designing the test part. The chosen design was based on canonical sphere-type geometry, which is a metrological ideal element widely used as a reference standard and easily definable as a best-fit entity in measurement software. The test part consists of nine spheres distributed in a three-by-three matrix (Figure 2). The sphere designation Sph X.Y corresponds to its position along the X and Y axes, taking the Sph 1.1 center as the origin. The spheres whose Y position is 1 have a 15 mm diameter, those in position 2 have a 10 mm diameter, and those in position 3 have a 5 mm diameter. Likewise, the X positions 1, 2, and 3 correspond to the location angles of 30°, 15°, and 0° (the reference is sphere centers), respectively. To avoid using support material in the SLM process, the spheres were designed with a 210° angle and located on the top of cylindrical bases, thus allowing the measurement of the upper hemisphere. These cylinders were joined by 3 mm thick walls to create a single part. Including spheres of different diameters and orientations was based on the metrological idea of evaluating the entire working volume of the SLM machine. The valid geometrical features of the part are the spheres; consequently, the contact and optical measurements were done on these entities.

### 2.2. SLM Manufacturing Process and Sandblasting Post-Process

A metal 3DSystems SLM ProX^®^ 100 machine was used to manufacture the sphere-based test part. It works with a 1070 nm, 38 W fiber laser. The scan speed and layer thickness parameters were set at 140 mm/s and 30 µm, respectively. Following the recommendations of the SLM machine manufacturer, the scanning strategy was defined as hexagonal. The maximum manufacturing volume is 100 × 100 × 100 mm with 20 µm repeatability in the three axes (X, Y, Z) and a typical accuracy of ±0.1%–0.2% (±50 µm minimum). During the manufacturing operation, the cabinet of the SLM machine was filled with nitrogen.

17-4PH stainless steel alloy was used for manufacturing the test part. It is a chrome–nickel–copper precipitation hardened stainless steel. Due to its high resistance and hardness properties, as well as its wear resistance, good corrosion resistance, and thermal properties up to 300°C, it is used in parts for different industrial sectors, such as the aerospace, chemical and petrochemical, energy, and medical–surgical sectors [37].

**Figure 3:**
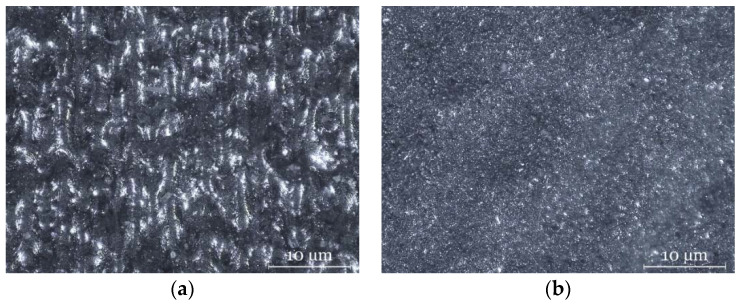
Microscope images at 92 × magnification: (**a**) pre-sandblasting SLM part; (**b**) post-sandblasting SLM part.

A sandblasting post-process is usually carried out after the SLM manufacturing. In this study, a Sablex S-2 machine with white aluminum oxide WFA F100 as abrasive material was used at 7 bar. On the one hand, sandblasting leaves the part with a matte surface finish that facilitates the 3D scanning process. On the other hand, this post-process removes the unmelted powder particles from the part, improving its surface roughness. Figure 3 shows the surface finish of the pre and post sandblasting SLM part (consider brightness and texture). The improvement of the surface finish also reduces measurement errors when the CMM stylus tip contacts the part surface.

### 2.3. Contact Measurement

A CMM DEA Global Image^®^ (Hexagon Metrology, currently known as Hexagon Manufacturing Intelligence, Hexagon AB, Stockholm, Sweden) was used to measure the test part with high precision, obtaining its GD&T measurements. According to ISO 10360-2:2009 [38], its maximum permissible error is MPEE (µm) = 2.2 + 0.003 L (L being in (mm)). Additionally, several recommended techniques were applied to compensate for the usual errors arising in CMMs calibration, such as inversion methods and several measurement repetitions.

For this study, the CMM was equipped with a contact scanning touch-probe, Renishaw SP25, and a 1 mm tip diameter. This tip size was chosen because of the surface texture that is due to the typically high roughness that characterizes SLM parts (Figure 3a). Despite the sandblasting post-processing (Figure 3b), these surfaces have irregularities and inclusions that make it difficult to capture points with contact systems due to their small size. These surface characteristics, among others, could induce errors and deviations in the measurement with different sensors when measuring the same SLM surface [13]. To minimize this error, the contact measurement was performed with a small diameter tip.

**Figure 4:**
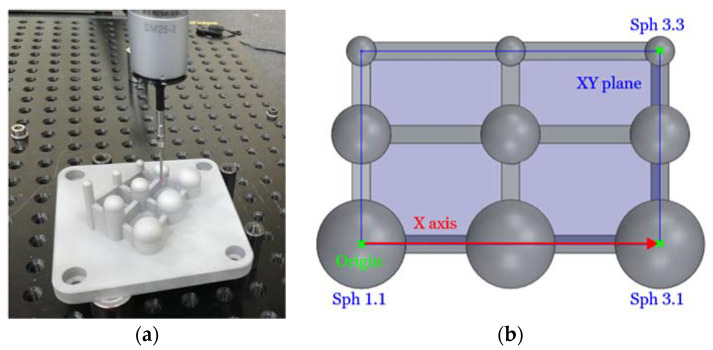
(**a**) Contact measurement of the test part; (**b**) alignment features of the test part.

Prior to CMM measurements (Figure 4a), the 3-2-1 alignment procedure was used using the XY plane constructed with the centers of Sph 1.1, Sph 3.1, and Sph 3.3 (which determines the + Z axis); the line constructed from Sph 1.1 center to Sph 3.1 center as the + X-axis; and the Sph 1.1 center as the origin (Figure 4b). After the first manual alignment, the part alignment was carried out in automatic mode, digitizing by contacting these three spheres (Sph 1.1, Sph 3.1, and Sph 3.3).

Considering that the main objective of this study is the evaluation of several OMSs, contact measurements (taken as reference) should be as similar as possible to optical scanning to obtain an accurate and real comparison. Based on the dense point clouds captured by these non-contact systems in previous measurements, a 2.55 points/mm^2^ density was estimated for the contact measurement. Table 1 shows the number of contact points in each sphere according to its diameter.

The GD&T dimensions were the diameters, form errors (sphericity), and center positions of the spheres. All the digitized data were processed using the computer-aided inspection (CAI) software PC-DMIS 2018 R2. In the 3D comparison, the scanned spheres and the quasi-real CAD model (obtained from the reference values obtained with the CMM) were used. Using this quasi-real CAD model minimizes the influence of manufacturing errors when making comparisons and best-fits between the actual spherical surfaces and the point clouds.

### 2.4. Optical Measurement

**Figure 5:**
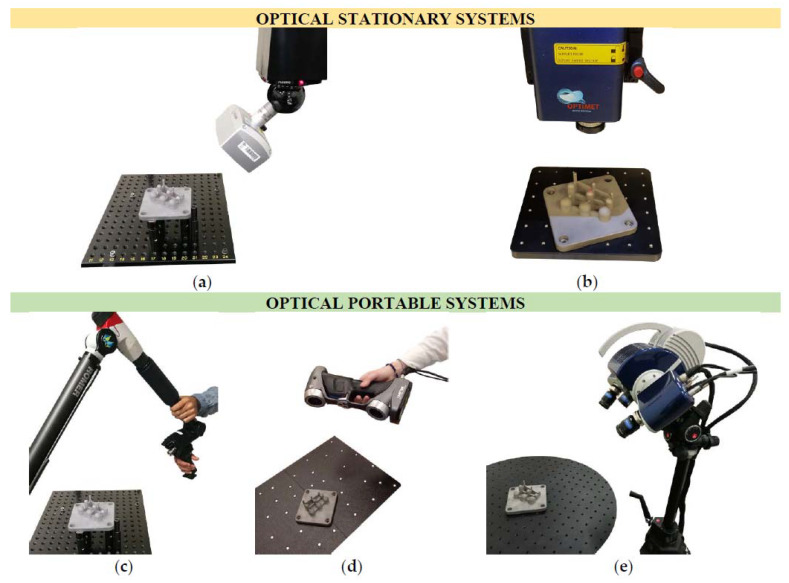
Optical measurement of the test part using stationary systems: (**a**) a laser scanner on a coordinate measuring machine (LS-CMM) and (**b**) a conoscopic holography (CH) sensor; and portable systems: (**c**) a laser scanner on a coordinate measuring arm (LS-CMA), (**d**) a handheld laser scanner (HLS), and (**e**) a structured light (SL) scanner.

Both contact and optical measurements were carried out under the same conditions and following the same alignment process. As mentioned earlier, this study focuses on optical measurements, more specifically on the evaluation and comparison of several OMSs, both stationary (LS-CMM and CH sensor) and portable (LS-CMA, LS, and SL scanner) (Figure 5). The following sections describe the systems and the measurement methodology used. For the five OMSs, the digitization process was defined to capture as much surface area of the spheres as possible for evaluating the form error.

#### 2.4.1. Laser Scanner on CMM (LS-CMM)

The CMM was a DEA Global Image^®^ with a motorized indexing head PH10-MQ (Renishaw). This head is capable of mounting several contact probes (TP20, SP25) and the newest generation of laser sensors, like the HP-L-10.6 Laser Scanning Sensor. This laser triangulation probe allows adapting the scan width according to the complexity of the surface and controlling the laser power in real-time (point by point) and automatically. The accuracy specifications of the sensor, which complies with ISO 10360-8:2013 [39], are a 34 µm probe dispersion value (P_Form.Sph.D95%:,MPL_) and a 22 µm probing form error (P_Form.Sph.1×25:,MPE_). Regarding data acquisition, the sensor has a 30,000 pts/s maximum data rate.

A scanning optimization process was carried out prior to the test part digitization to determine the optimum number of scans (passes) and laser head orientations. For it, a 19.9929 mm diameter, optically suitable (made of white matte ceramic) and calibrated sphere was scanned using a different number of passes with different probe head orientations: from a single one pass (vertical 0°) to five passes (vertical and four cardinal points with a 45° angle). This last method was finally selected because it covers the entire upper hemisphere and even a small area below the equator, ensuring a correct degree of coverage and without loss of precision. Each of these five probe head orientations was calibrated with the same laser setup parameters: a 16.8 pts/mm laser-line points density and a 123 mm laser width. This laser setting is considered the most suitable for the test part measurement, allowing the capture of points over the nine spheres in every single scan for a given laser head orientation. Following manufacturer recommendations to increase the scanning accuracy, all scans were made using a 75° incidence angle filter, which means that only those points with a normal direction between 75° and 90° were acquired. This filter was applied in real-time during the scanning process (Figure 5a).

#### 2.4.2. Conoscopic Holography Sensor (CH)

The ConoScan 4000 (Optimet) is a CH system that uses sensors with interchangeable objective lenses. In this study, a ConoProbe sensor with a 100 mm objective lens was used to provide a capture range capable of containing all the part spheres despite the different heights and diameters. The main specifications of the CH sensor are a 15 µm Z precision, a 170°angular coverage, and a 50 mm/s maximum travel speed. Unlike the rest of the available systems, in which the orientation of the scanning head can be configured, in the ConoScan 4000, only movements perpendicular to the XY plane (a mobile platform where the part to be scanned is attached) can be made. The parameter settings were selected to capture a uniform point cloud similar to that obtained with the LS-CMM system. To achieve this, a step was selected along the lines of 0.059 mm and between lines of 0.06 mm. Regarding the scanning parameters, a 1400 W laser power and 1500 Hz frequency were defined. Together with the CH sensor, the ConoScan 4000 software was used for measuring the test part (Figure 5b).

#### 2.4.3. CMA with Integrated Laser Scanner (LS-CMA)

The Romer Absolute Arm 7525 SI (Hexagon Manufacturing Intelligence) is a CMA with a fully integrated and certified RS3 laser scanner. The scanning sensor specifications, according to ISO 10360-8:2013 [39], are a 30 µm accuracy and a 460,000 pts/s maximum point acquisition rate. The LS-CMA is a manual process where the operator controls the scanner mounted on a portable arm. Considering the manual nature of this process, there is variability among measurements, even if the process is performed by the same operator [28]. Nevertheless, when the measurements are taken by different operators the dispersion of values is greater than when the same operator takes several repetitions. Therefore, considering this influence, the test part was scanned by five different operators who made two measurements each. The digitization of the test part (Figure 5c) with the LS-CMA was performed by capturing as many points as possible according to the five orientations defined in the scanning methodology, as mentioned for the LS-CMM equipment. PolyWorks^®^ software was used for controlling the LS-CMA and for generating the raw point clouds.

#### 2.4.4. Portable and Handheld Laser Scanner (HLS)

The HandySCAN700™ (Creaform Inc., Lévis, QC, Canada) is a portable and handheld laser triangulation scanner designed for RE and metrology applications, offering high-precision measurements. Its main specifications are a 50 µm resolution of a 20 µm + 60 µm/m volumetric accuracy. Regarding the scanning parameters, this sensor works with seven transversal red lasers (+1 additional line) and has a measurement speed of 480,000 pts/s. Despite being a manual system, the variation among measurements made by different operators, and even those made by the same operator at different repetitions, is negligible due to the automatic filtering scanning mode that avoids cloud overlaps. The maximum deviation for this scanner was 15 µm when measuring the diameter of the spheres by different operators. Therefore, only one optical measurement (Figure 5d) was carried out for this study using the HLS system and the VXelements™ software.

#### 2.4.5. Structured Blue-Light Scanner (SL)

The Breuckmann smartSCAN^3D^-HE (currently known as the AICON SmartScan) is a structured blue-light scanner based on the fringe pattern projection technique. It works based on the miniaturized projection technique. Depending on the characteristics of the object to be measured, an appropriate sequence of blue-light fringe patterns is displayed by the projection unit onto the part. This unit has a 28 Mpx resolution and 550 ANSI Lumen. The acquisition system consists of two-cameras with a 4 Mpx resolution (per camera) and captures the projected fringe pattern at a predefined viewing angle according to the selected field of view (FOV). The size of the FOV is determined by the diagonal length of the measurement volume. A 125 mm FOV was used for this study since it is the smallest FOV that allows capturing the entire volume of the test part. The main specifications of the SL scanner are a 50 µm X and Y resolution, a 5 µm Z resolution limit, and a 9 µm feature accuracy.

The test part was digitized after the SL scanner calibration operation. Data acquisition was carried out using the OPTOCAT software set in automatic measurement mode (used in combination with a turntable). Six scans were performed for the entire digitization of the test part, so the turntable moved 60° between each one. Figure 5e shows the SL scanner during the test part scanning process.

### 2.5. Treatment of Point Clouds and GD&T Measurements

All point clouds resulting from the optical scans were imported in Geomagic^®^ Control X™ inspection software as ASCII files (*.asc format). In the first step, all points included in the raw point clouds that do not belong to the part spheres were removed. The cleaning operations also include manual removal of points placed under virtual planes. These planes were located 1 mm above the intersection between the sphere and the cylinder that supports it (for each entity). The test part was always aligned in the same way for the different point clouds generated by the evaluated OMSs for later comparison. According to the CMM alignment (Section 2.3), the spheres Sph 1.1, Sph 3.1, and Sph 3.3 were used to align the point clouds with respect to the quasi-real CAD. Finally, the nine scanned spheres were reconstructed as measurement features. The virtual spheres generation was done using the “best-fit” adjustment method (least squares algorithm) for each evaluated OMS using its own control software. The laser triangulation (LS-CMM, LS-CMA, and HLS) and SL scanners allow scanning almost the entire spheres’ surface. However, in the case of the CH sensor, due to the vertical position of the sensor in relation to the part (its unique movement is in the XY plane), the resulting point cloud does not cover the spheres completely, so points located on the equator were not captured.

A single point cloud was generated for each system (LS-CMM, CH, HLS, and SL) except for the LS-CMA. In this case, due to the high variability of the results by “manual” scanning, it was necessary to generate ten point clouds. The average values obtained from these ten repetitions were taken as representative of this system for each sphere.

In the next step, the dimensional and geometrical values of the spheres were evaluated using the reconstructed features or the virtual spheres. Finally, a 3D comparison was made between the quasi-real CAD and the virtual spheres obtained with the different OMSs.

## 3. Results and Discussion

The most relevant results are presented in this section. The main objective of this work is to compare the accuracy of several OMSs for inspecting SLM parts by scanning. Additionally, this comparison was extended to also consider the processing time for all the cycles, due to the great differences that exist for the OMSs. Other factors such as cost, difficulty/ease of use, or operator involvement were not considered in this study since they are hardly quantifiable attributes.

### 3.1. GD&T Comparison among the Evaluated OMS

As mentioned earlier, all the spheres were scanned, trying to capture the maximum possible sphere surfaces, not only above the equator but even slightly below, ensuring the capture of points on the sphere equator and along its entire length (apart from the CH sensor, where the equator is the capture boundary).

**Figure 6:**
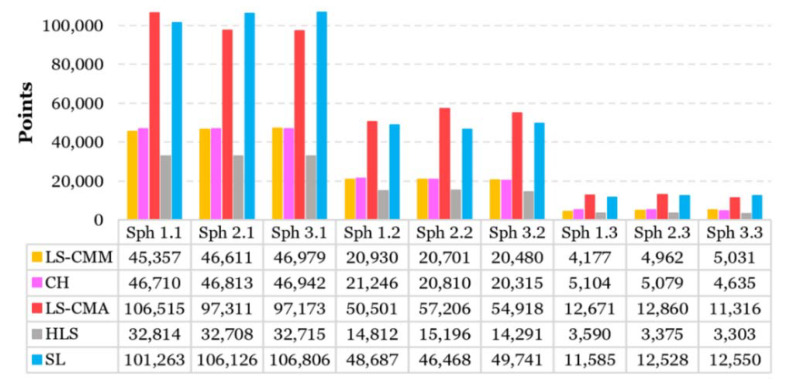
Number of points used in the creation of control spheres for the different optical measurement systems (OMSs).

Each system captured a different number of points. The number of points used to create the control spheres is shown in Figure 6 after performing the point cleaning operation. In all the spheres, the number of points captured by the LS-CMA and the SL scanner is about twice those obtained by the rest of the OMSs (Figure 6). This is due, in the case of the SL scanner, to the high point acquisition capacity and, in the case of the LS-CMA, to the several scans (passes) performed by the operator to capture the entire surface of the spheres. The least dense point cloud was obtained by the HLS system. This is due to the capturing process of this equipment, which does not allow overlapping points in areas that have already been captured.

The results in the next section, relative to the dimensional and geometrical measurement errors, were calculated from the reference values obtained with the CMM (quasi-real CAD values).

#### 3.1.1. Dimensional Errors: Diameter and Spheres Center Position

**Figure 7:**
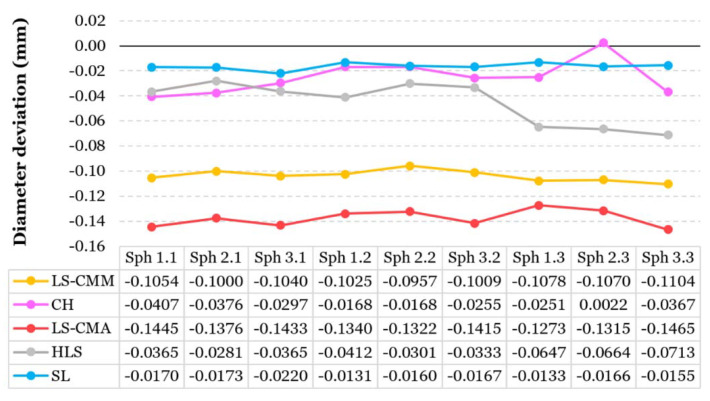
Deviation of sphere diameters for the optical measurements (OMS) from the reference values (CMM).

Dimensional errors that result from measuring the sphere diameters present a horizontal trend (similar values) regardless of the sphere size for LS-CMM, LS-CMA, and SL systems (Figure 7); that is, these errors are associated with the system and not with the entity dimension. These OMSs have average deviation values of 0.1037 mm (LS-CMM), 0.1376 mm (LS-CMA), and 0.0164 mm (SL). In the case of the HLS scanner, the obtained deviations were higher for the smaller diameter spheres (Figure 7), which implies a decrease in accuracy for smaller geometries. The CH system has a wide variation among its measurements (without following a clear trend), ranging from 0.0022 mm to 0.0407 mm. As shown in Figure 7, the equipment with the least deviations from the CMM contact measurements is the SL scanner. The system with the highest deviations is the LS-CMA followed by the same laser scanning technology installed on a CMM (Figure 7).

Regarding the position of the sphere centers, in general, the highest deviations occur for the smaller diameter spheres (Sph 1.3, Sph 2.3, and Sph 3.3). The three laser triangulation systems and the SL scanner have the highest deviations in the Z coordinate (Table 2). Conversely, the systems with the least deviations were the HLS and SL scanners (Table 2). Unlike the rest, the CH system has higher deviations in the X coordinate and lower in the Z coordinate (Table 2). This is mainly because, in this system, the scanning movement is performed in the X direction, without a simultaneous displacement of Y or Z axes. The deviation also increases both with the reduction in sphere diameter and with the increase in the location height of the spheres.

#### 3.1.2. Geometrical Errors: Sphericity and Standard Deviation

**Figure 8:**
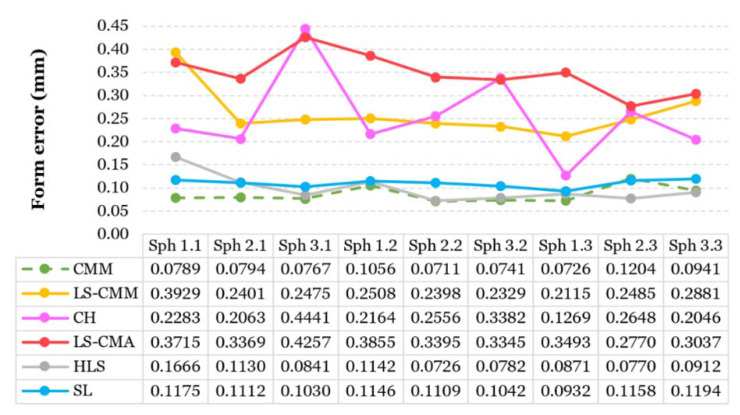
Contact and optical measurements of sphere form error.

Figure 8 shows the spheres form error for each measurement system. The two OMSs that come closest to the reference values obtained by contact are the HLS and SL (Figure 8), whose sphericity values are around 0.0982 mm and 0.1100 mm, respectively. As shown in Figure 8, the system with the highest form error values is the LS-CMA, with an average value of 0.3471 mm. The CH sensor has an irregular behavior with large variability in the results, reaching a maximum form error value of 0.4441 mm and a minimum of 0.1269 mm (Figure 8).

**Figure 9:**
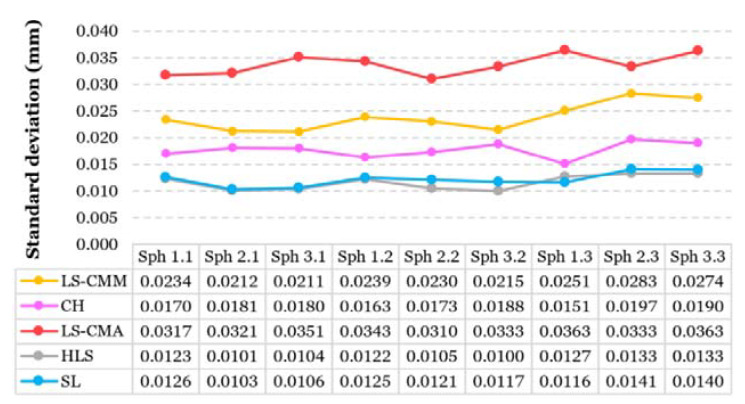
Standard deviation of the point clouds from the reference values (CMM).

Optical systems generate spurious points during the digitization of parts with reflexive or irregular surfaces. This effect can alter the results of form errors. Therefore, it is preferable to not use the value of the “metrological” sphere form error (ISO 1101:2017 [40]) for measuring the quality of the point clouds. Instead, in this work, we have used the value of the point cloud standard deviation. This parameter is much more representative of the point cloud quality, reducing the enormous influence that a few spurious points can have on the value of the form error. Figure 9 shows the standard deviation of the resulting point clouds for each sphere and the different evaluated OMS. As can be seen, the HLS and SL scanners have the lowest standard deviation, with an average value of 0.012 mm in both systems.

#### 3.1.3. 3D Comparison between the Evaluated OMS and the Quasi-Real CAD

**Figure 10:**
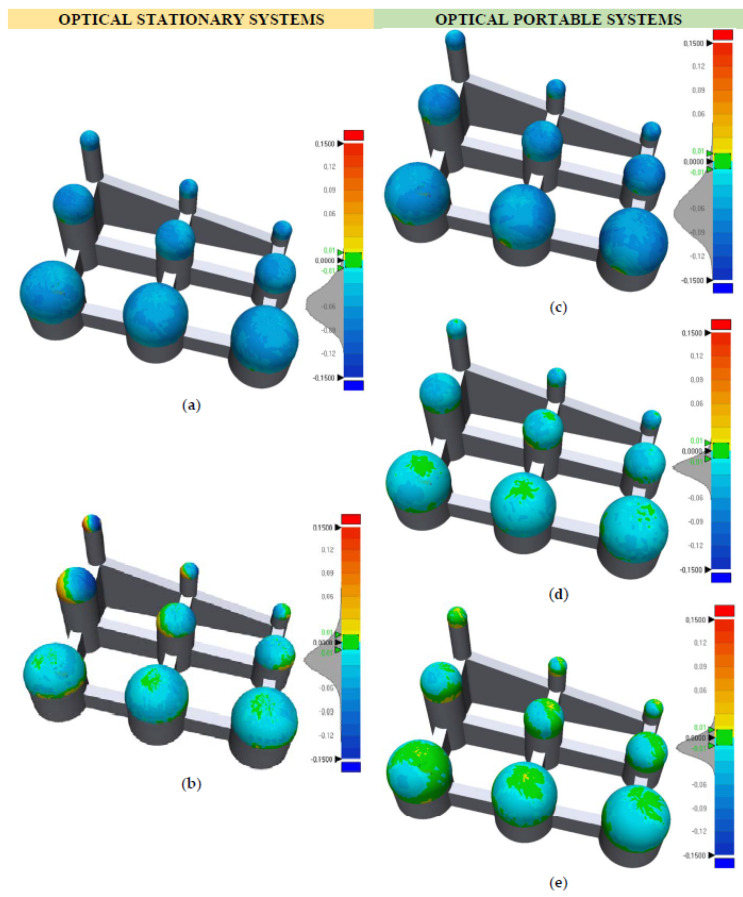
3D comparison between the quasi-real CAD and the point clouds of the OMS: (**a**) LS-CMM; (**b**) CH; (**c**) LS-CMA; (**d**) HLS; (**e**) SL.

The 3D comparison between the optical systems and the quasi-real CAD (Figure 10) shows the results of the geometrical and dimensional errors analyzed in the previous sections (Section 3.1.1 and Section 3.1.2).

As before, the results closest to the reference values are those obtained by the portable HLS (Figure 10d) and SL (Figure 10e) systems. The SL scanner had the smallest deviations, with an average value of 0.0116 mm and a standard deviation of 0.0123 mm (Table 3). The most unfavorable results are those obtained by the LS-CMM (Figure 10a) and LS-CMA (Figure 10c) scanners, whose average deviations are 0.0561 mm (with a standard deviation of 0.0230 mm) and 0.0611 mm (with a standard deviation of 0.0311 mm), respectively (Table 3). As described in Section 3.1.1, the CH system presents a displacement of the coordinate origin on the X-axis based on the distance to the sensor, leading to greater deviations with the increase in the location height of the spheres (Figure 10b).

### 3.2. Comparison of the Complete Inspection Process Time

**Figure 11:**
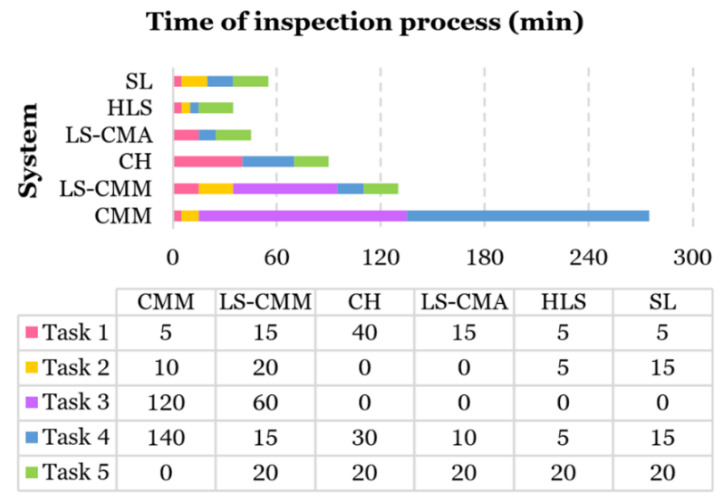
Approximate time spent on each task for each measurement system (contact and optical).

One of the most relevant aspects for companies is the processing time. Less process time means higher production and, therefore, more economic benefit. Digitization is only a part of the entire inspection process and, therefore, conveys a portion of the total time required for inspecting the part. Taking this aspect into account, a comparison of the full time employed during the inspection process was performed for each measurement technology (contact and optical) (Figure 11). The inspection time was divided into five main tasks:

- Task 1—Setup: Setups consist of switching on both the measurement equipment and software. In the case of non-contact systems, this activity also includes optical sensor heating.
- Task 2—System calibration: The CMM was calibrated using a certified calibration sphere measured by contact. The LS-CMM was optically calibrated using a specific laser calibration sphere. The HLS and SL scanner calibrations were carried out by measuring a calibration plate (marked with circles). The rest of the systems do not have a specific calibration task; the calibration is integrated into the equipment setup once started.
- Task 3—Program preparation: This activity is only necessary when the measurement software is PC-DMIS. It consists of editing the measurement program, including the output variables that will compose the results report.
- Task 4—Execution of digitization: This task consists of acquiring data to obtain the dimensional and geometrical information of the test part. For the CMM, a manual pre-alignment is done first, then an automatic alignment and measurement are carried out. In the case of the LS-CMA and HLS systems, this task is done manually, whereas, for the rest of the OMSs, it is done in automatic mode. This activity also includes setting scanning parameters without requiring program preparation.
- Task 5—Generation of results report: The results report generation is automatic in the case of the CMM with PC-DMIS software. For the non-contact systems, this activity is performed with the Geomagic^®^ Control X™ software, and it includes the tasks of point cloud cleaning, sphere reconstruction, and generating the results report.

The time spent in the process changes depending on the type of inspection. In the case of a single-part inspection, the time spent will correspond to the total time (Figure 11). However, if a serial inspection is carried out (several parts), the time will correspond to the time spent in the first three tasks plus the time in Tasks 4 and 5 multiplied by the number of parts (Figure 11). In addition, if it is necessary to perform the inspection of another part out of the lot afterward, the time spent will be the total time minus the time spent on Task 3 (Figure 11).

As expected, for a single inspection of the test part, the quickest measurement systems are the three portable scanners (LS-CMA, HLS, and SL). In this case, the full inspection process can be performed in less than 1 h (Figure 11). In the case of serial inspection, the quickest systems are the three portable systems together with the LS-CMM (since the time spent in Task 3 is dispensed with in this case). When the objective is to use the OMS equipment for indistinctly measuring one or many parts, the most advantageous equipment is the HLS and the least advantageous is the CMM (Figure 11), although, undoubtedly, this is the most accurate equipment.

## 4. Conclusions

The influence on the measurement results of optical inspection using different metrological systems and technologies is analyzed in this study. This study is based on evaluating five OMSs with different characteristics and working principles: CH, SL, and three different sensors based on the laser triangulation principle. Two of them are stationary (LS-CMM and CH) and the other three portables (LS-CMA, HLS, and SL). The major contribution of this work with respect to other studies is the test part, designed specifically for this study, and the application to a relatively modern AM process—the SLM process for metallic parts. In this particular case, a sphere-based test part made of 17-4PH stainless steel was designed, manufactured, measured, and analyzed. The special characteristics of this type of parts made by AM, the new trends in dimensional metrology, and the search for higher speeds and precision in inspection tasks make this type of study necessary. Considering that the main objective of the research is analyzing the dimensional and geometrical accuracy of the different optical measuring systems for verifying parts manufactured by SLM, our sphere-based test part was designed to allow enough accessibility of both contact and optical systems. The main findings of this study may be summarized as follows.

In terms of inspection speed, regardless of the number of parts, portable systems are the most advantageous. Likewise, the HLS and SL portable systems had the lowest dimensional deviations (diameter and center position of the spheres) and geometrical deviations (form error and standard deviation); that is, their results were the closest to the reference values (obtained with a CMM by contact). Either of these two OMS is suitable for measuring SLM parts (characterized by a typical high roughness). The extreme portability, lightness, speed, and ease of handling give the HLS system great advantages in a wide range of industrial applications. However, for high-precision measurement of smaller geometries, the most suitable system is the SL.

This initial comparison enables to qualify the different optical technologies, considering the special finish of the SLM-manufactured parts. Based on the results of this comparison, our group is currently working with specific technologies using test parts containing more geometrical features (including sharp corners and concave features at different orientations), without considering the extensive range of technologies studied in this paper. This will allow analyzing the suitability of the systems identified in this paper as the best ones, incorporating aspects of accessibility. In addition, the design of this type of extended feature-based test parts will allow analyzing the dimensional and geometrical accuracy achieved in SLM additive processes.

## Figures and Tables

**Table 1:** Contact measurement parameters depending on the sphere diameter.

Visualization	Spheres	Diameter (mm)	Semi-Sphere Area (mm^2^)	Contact Points	Density (Points/mm^2^)
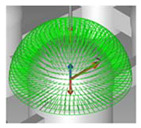	Sph 1.1 Sph 2.1 Sph 3.1	15	353	900	2.55
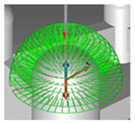	Sph 1.2 Sph 2.2 Sph 3.2	10	157	400	2.55
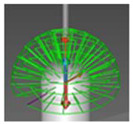	Sph 1.3 Sph 2.3 Sph 3.3	5	39	100	2.55

**Table 2:** Average deviation of the sphere’s center position according to the X, Y, and Z axes of the optical measurements from reference values (CMM).

Parameter		LS-CMM	CH	LS-CMA	HLS	SL
Deviation of sphere center position (mm)	X	0.0011	−0.0369	−0.0028	−0.0018	−0.0011
	Y	0.0042	−0.0056	0.0039	−0.0015	−0.0020
	Z	0.0056	−0.0049	0.0078	0.0062	0.0083

**Table 3:** Average, standard deviation, and RMS of the 3D comparison between the quasi-real CAD and the point clouds of the OMS.

Parameter	LS-CMM	CH	LS-CMA	HLS	SL
Average (mm)	−0.0561	−0.0224	−0.0611	−0.0220	−0.0116
Standard Deviation (mm)	0.0230	0.0279	0.0311	0.0125	0.0123
RMS (mm)	0.0607	0.0358	0.0686	0.0253	0.0169
